# Occurrence and Distribution of Antibiotics in a Tropical Mariculture Area of Hainan, China: Implications for Risk Assessment and Management

**DOI:** 10.3390/toxics11050421

**Published:** 2023-05-01

**Authors:** Yongqiang Qin, Xiaoyü Ren, Hanye Ju, Yankun Zhang, Jin Liu, Jiliang Zhang, Xiaoping Diao

**Affiliations:** 1 Ministry of Education Key Laboratory for Ecology of Tropical Islands, Hainan Normal University, Haikou 571158, China; 2 College of Life Science, Hainan Normal University, Haikou 571158, China; 3 College of Ecology, Environment Hainan University, Haikou 570228, China; 4 Hainan Research Academy of Environmental Sciences, Haikou 571126, China

**Keywords:** antibiotics, tropical mariculture area, distribution, ecological risk

## Abstract

With the rapid global demand for mariculture products in recent years, the use of antibiotics has increased intensively in the mariculture area. Current research on antibiotic residues in mariculture environments is limited, and less information is available on the presence of antibiotics in tropical waters, limiting a comprehensive understanding of their environmental presence and risk. Therefore, this study investigated the environmental occurrence and distribution of 50 antibiotics in the near-shore aquaculture waters of Fengjia Bay. A total of 21 antibiotics were detected in 12 sampling sites, including 11 quinolones, 5 sulfonamides, 4 tetracyclines, and 1 chloramphenicol; the quinolones pyrimethamine (PIP), delafloxacin (DAN), flurofloxacin (FLE), ciprofloxacin (CIP), norfloxacin (NOR), pefloxacin (PEF), enrofloxacin (ENO), and minocycline (MNO) of the tetracycline class were detected in all sampling points. The total antibiotic residue concentrations in the study area ranged from 153.6 to 1550.8 ng/L, the tetracycline antibiotics were detected in the range of 10 to 1344.7 ng/L, and the chloramphenicol antibiotics were detected in the range of 0 to 106.9 ng/L. The detected concentrations of quinolones ranged from 81.3 to 136.1 ng/L, and the residual concentrations of sulfonamide antibiotics ranged from 0 to 313.7 ng/L. The correlation analysis with environmental factors revealed that pH, temperature, conductivity, salinity, NH^3−^-N, and total phosphorus had a strong correlation with antibiotics. Based on PCA analysis, the main sources of antibiotic pollution in the area were determined to be the discharge of farming wastewater and domestic sewage. The ecological risk assessment indicated that the residual antibiotics in the water environment of the near-shore waters of Fengjiawan had certain risks to the ecosystem. Among them, CIP, NOR, sulfamethoxazole (TMP), ofloxacin (OFL), enrofloxacin (ENO), sulfamethoxazole (SMX), and FLE showed medium to high risk. Therefore, it is recommended to regulate the use of these antibiotics and the discharge and treatment of culturing wastewater, and measures should be taken to reduce the environmental pollution caused by antibiotics and to monitor the long-term ecological risk of antibiotics in the region. Overall, our results provide an important reference for understanding the distribution and ecological risk of antibiotics in Fengjiawan.

## 1. Introduction

As one of the most important medical discoveries of the 20th century, antibiotics are widely used to treat diseases and promote animal growth in many fields, such as livestock, aquaculture, and agriculture [1,2]. China is the largest producer and consumer of antibiotics in the world, with an estimated production of 210,000 tons in 2007 and 248,000 tons in 2013 [3]. In 2013, China used about 162,000 tons of antibiotics, with veterinary consumption accounting for about 52%, of which fluoroquinolones APIs, sulfonamides, and macrolides accounted for 48% of the total consumption [4]. Global antibiotic consumption increased by 65% between 2000 and 2015, in addition to doubling the consumption of veterinary antibiotics in China by 2030 [5]. Therefore, the frequent detection of these chemicals has been in the aquatic environment in China [6].

The nearshore sea is the region where the biosphere, atmosphere, and hydrosphere intersect frequently and with the most intense activities and is closely related to the survival and development of human beings. The unique geographical environment of the nearshore sea has led to the rapid development of human activities as well as aquaculture industries. Due to rapid economic development and extensive anthropogenic activities, water pollution has become a key pressure affecting ecologically vulnerable areas in nearshore seas [7,8]. The ocean is a sink for environmental pollutants, and antibiotic contamination is prevalent in the global ocean, such as the Yellow Sea [9], Bohai Bay [10,11], South China Sea [12,13,14], the East Sea of China [15], San Francisco Bay of the United States [16], the southern Baltic Sea [17], and Cadiz Gulf of Spain [18]. In 2014, more than 110 drugs or their metabolites were detected in global offshore waters, of which more than 40 belonged to the antibiotic class, mostly in the range of 1 ng/L to several thousand ng/L, with sulfonamides, quinolones, tetracyclines, and chloramphenicol are the most common [19]. In the same year, clarithromycin CLM, erythromycin ETM, roxithromycin RTM, and SMX residues were detected in 153 seawater samples from nearshore waters of Germany, Italy, Greece, and the United States, with SMX being detected most frequently in San Francisco Bay at concentrations ranging from 13 to 61 ng/L [16]. Siedlewicz [17] detected seven antibiotics in the Baltic Sea, with the highest level detected for SMX at 311 ng/L. Kim [20] detected nine antibiotics in Korean waters, with the highest level of lincomycin detected at 438 ng/L. McEneff [21] detected antibiotics in seawater in the UK at concentrations ranging from 70 to 870 ng/L. In addition, antibiotic detections have also been reported in the Mediterranean Sea [22], Red Sea [23], Belgian inshore waters [24], and Spanish Cadiz Gulf [18]. Compared to other countries, antibiotics pollution in China is more severe, and some research on antibiotics has been conducted in the last decade on Bohai Bay, Beibu Gulf, Jiaozhou Bay, Laizhou Bay, Yantai Bay, Liaodong Bay, and Shenzhen Bay [25,26,27]. Moreover, researchers have investigated antibiotics contamination at an area of 18,000 km nearshore sea and found their residue levels in the range of 389 to 3302.3 ng/L [28]. Despite a large number of previous studies, limited information is available on antibiotic residues in mariculture environments in tropical regions, which limits a comprehensive understanding of their environmental presence and associated risks. 

Antibiotic residues are a potential risk to both ecosystem and human health and can cause life-threatening complications for humans and animals [29]. For example, antibiotics could decrease the plankton and algae population, affect the behavior of aquatic organisms, and alter the aquatic community structure [30]. Meanwhile, sulfamethoxazole (SMX) and enoxacin (ENX) can be biomagnified in marine organisms and may pose adverse effects on human health via food chain transfer [31]. It has been shown that antibiotics are usually not completely absorbed or metabolized by humans or animals, with approximately 60% to 90% of antibiotics not being absorbed and metabolized by animals but excreted as prodrugs or metabolites [32]. At least 30% of antibiotics enter aquatic ecosystems via agricultural activities, animal manure discharge, sewage discharge, or surface runoff [33,34,35] and eventually flux to the nearshore marine environment through river inputs and surface runoff, altering the community structure of marine ecosystems, changing or even stopping the material cycle of marine ecosystems, reducing the species diversity of seawater, destabilizing marine ecosystems, and threatening the survival of organisms. In addition, long-term antibiotic abuse induces the production of antibiotic resistance genes (ARG) in animals and microorganisms, contributing to the widespread dissemination of ARGs in the environment. The World Health Organization reported that antibiotic resistance caused by antibiotics has become a major global public health security issue threatening human health. Drug-resistant bacterial infections are reported to cause more than 700,000 deaths globally each year. By 2050, drug-resistant bacterial infections will cumulatively cause 10 million deaths worldwide [36]. Therefore, there is a need to understand the occurrence and ecological risk of antibiotics in the environment.

In this study, we used liquid chromatography-tandem mass spectrometry (LC-MS/MS) to quantify 50 antibiotics in four major classes (sulfonamides, quinolones, chloramphenicol, and tetracyclines) in water samples from the offshore waters of Fengjia Bay in tropical China. Combined with the sampling environment, we used PCA analysis to find the possible sources of antibiotic input in the region and reveal the environmental factors influencing antibiotic contamination. We also used an ecological risk model to assess the ecological risk of residual antibiotics in the region, to improve our understanding of the geochemical processes and ecological risks of these chemicals, and to provide useful information for the development of risk management strategies for antibiotic pollution in the offshore aquaculture area of Fengjiawan, Wenchang.

## 2. Materials and Methods 

### 2.1. Experimental Materials and Equipment

A total of 50 antibiotic standards for four major classes of antibiotics (quinolones, sulfonamides, chloramphenicol, and tetracyclines) were obtained from Beijing Manhag Biotechnology Co., Ltd. (Beijing, China). Other chemical reagents and equipment used in this study were Na_2_EDTA (analytical purity, Guangzhou Chemical Reagent, Haizhu District, Guangzhou, China), formic acid (HPLC grade, ROE SCIENTIFIC INC), methanol (HPLC grade, Honeywell, Shanghai Honeywell Trading Co., Ltd., Shanghai, China), acetonitrile (HPLC grade, ThermoFisher, Thermo Fisher, Waltham, MA, USA), ultrapure water (Cascade Lab Water Systems, resistivity 18.2 mΩ/cm), ammonia (analytical purity, Shanghai Test, Sinopharm Ltd., Shanghai, China), 6 M HCl (analytical purity, Xilong Science Co., Ltd., Shantou, Guangdong, China), hydrophilic and lipophilic solid phase extraction Oasis HLB column (Watford Waters, UK), Microporous Filtration Membrane (Jinteng, China), YSI Water Quality Analyzer (3074, USA), liquid chromatograph—triple quadrupole tandem mass spectrometer (AB SCIEX Triple Quad6500+, USA), fully automatic solid phase extractor (Sheng Kang E9000, China), 1 mL syringe, needle filter membrane (biosharp, Shanghai, China), 50 mm diameter 0.22 μm pore size microporous filter membrane (Zinten, China) Water Purifier (Cascade Lab Water Systems, Barnsley, UK), Electronic Balance (Mettler, Switzerland), Nitrogen Blowing Instrument (DCY-12G Haikou, Haikou, China), pH Meter (PHSJ-4F Raycom, Shanghai, China), and Ultrasonic Cleaner (SK7200HP KUDOS, Shanghai, China).

### 2.2. Sample Collection 

**Figure 1:**
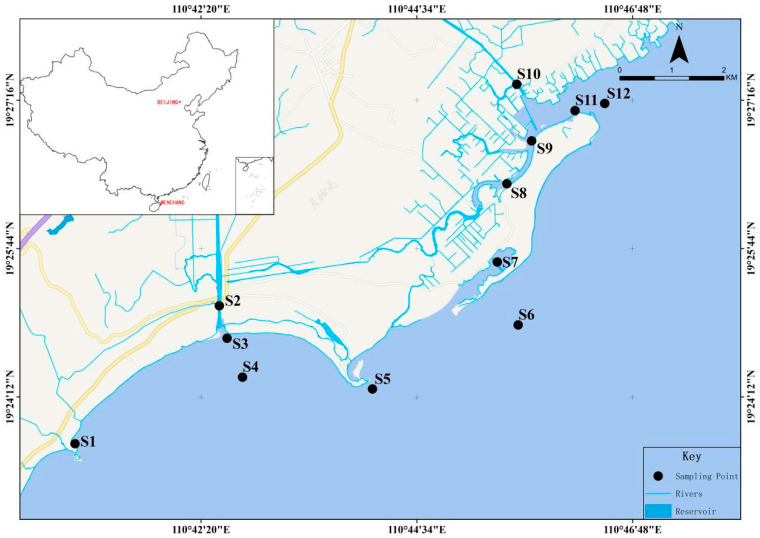
Sampling locations of surface water in Wenchang.

Wenchang Fengjiawan is located on the eastern coast of Hainan, with a length of about 15 km and a width of 1~4 km; as an important breeding area in Hainan Province, Wenchang Fengjiawan is surrounded by the most important Dongfeng snail breeding and shrimp fry breeding bases in Huewen Town, and the output value of shrimp fry used to account for more than 70% of the output value of Hainan and about 30% of the country. After decades of development, the Fengjiawan seawater aquaculture industry gradually formed a scale, mainly breeding farming grouper, Dongfeng snails, etc., but also farming South American white shrimp, factory farming, and pond farming, the number of farmers reached a thousand, and farming water surface of more than 10,000 acres. Farmers use a long pumping pipe to the middle of the sea to extract water from the sea floor to the home breeding pool and then use a long drainage pipe to flux the wastewater back to the sea. Surface water was sampled from 12 sites in Fengjiawan, Wenchang (Figure 1). Sampling sites were chosen to rationally reflect the water quality distribution statutes of Fengjiawan, including the sections that were greatly affected by domestic sewage, river inlets, nature reserves, fishing boat moorings, and aquaculture areas. Sampling sites S1–S4 were located in Wenchang Xincun Port; sites S5–S7 were located in Wenchang Bianhai Village Management Area; sites S8–S9 were located in Wenchang Changqi River; sites S10–S12 were located in Wenchang Changji Port. Sampling was conducted in April 2021. The water temperature (T), salinity (SAL), dissolved oxygen (DO), and pH of the water samples were measured in situ using a portable YSI Water Quality Analyzer (3074, USA). Total phosphorus (TP), ammonia nitrogen (NH^3−^-N), and chemical oxygen demand (COD) were measured by Guowei Yike Environmental Ltd. in accordance with the standard method of the State Environmental Protection Administration (SEPA) of China (SEPA, GB17378.4-2007). All samples were collected in triplicate from each site, kept in an ice box, and transported to the laboratory for immediate treatment. 

### 2.3. Sample Extraction 

The antibiotics in the water samples were concentrated by hydrophilic and lipophilic solid phase extraction Oasis HLB column ((500 mg, 6 mL, Watford Waters, UK). Before the extraction, a specific volume of water sample (3 L) was filtered through 50 mm diameter and 0.22 μm pore size microporous filter membrane (China, Jinteng). Aqueous samples of antibiotics were pretreated by adding 0.25 g of Na_2_EDTA per bottle, and 50 ng each of three internal standards (ofloxacin, sulfadiazine and ciprofloxacin) were added to each bottle to control the accuracy of the experiment. Pretreatment of quinolone antibiotics was done according to our own optimized pretreatment method for quinolone antibiotics, and sulfonamides, chloramphenicol and tetracycline antibiotics were done according to the optimized pretreatment method in the published article [34,35,36]. This was performed as follows: the pH of the filtered water sample was adjusted to 10 [37] with ammonia for quinolone antibiotics and 6.0 M HCl for sulfonamides, chloramphenicol, and tetracycline antibiotics; the water sample was extracted using an automatic solid phase extractor, and the HLB column was firstly washed with 20 mL of methanol followed by 6 mL of pure water; the quinolone antibiotics were filtered at a rate of 10 mL^.^ min^−1^, and 6 mL·min^−1^ for sulphonamides, chloramphenicol, and tetracycline antibiotics. When the sample was filtered, nitrogen was blown under a vacuum to dry the water on the HLB column, and the HLB column was eluted with 10 mL of methanol. The tube containing the eluate was removed, nitrogen was blown to near dryness, a fixed volume of 1 mL of methanol eluate was used, and then the extracted sample was transferred through a needle membrane filter into a brown injection vial using a syringe and assayed on a machine.

### 2.4. Instrumental Analysis 

The target antibiotics were analyzed via liquid chromatography-tandem mass spectrometry (HPLC–MS/MS). The HPLC separation was performed using a SCIEX 6500+ series (AB SCIEX Triple Quad6500+, USA) equipped with an a00B-4723-Y0 LC column (50 mm × 3.0 mm, 2.6 μm). The column was maintained at 40 °C during the sample analysis. The mobile phase consisted of eluent A (0.1% formic acid in ultrapure water) and eluent B (0.1% formic acid in acetonitrile). The flow rate was kept at 0.3 mL·min^−1^, and the injection volume was 10 μL. The separation of antibiotics was achieved with a gradient program as follows: starting with 3% mobile phase B for 1 min, then linearly changing from 3% mobile phase B to 95% mobile phase B within 13.5 min.

Mass spectrometry ion source was an electrospray ion source. Sulfonamides, quinolones, and tetracyclines antibiotics were collected in positive ion mode. Chloramphenicol antibiotics were collected in negative ion mode and detected in multi-reaction ion scan mode. The nebulizer pressure was set to 50 psi, the nozzle voltages were 5000 V. The flow rate and temperature of the sheath gas were 8 L·min ^−1^ and 550 °C, respectively. Quantification of each target compound was performed in the MRM mode. 

### 2.5. Quality Control and Quality Assurance 

All data generated from the analysis were subject to strict QA/QC procedures. Solvent and procedure blanks were simultaneously run in sequence to eliminate background noise and monitor system performance. In this study, all antibiotics were quantified by an external standard method. The standard curves for each antibiotic were obtained by gradient dilution of the standard sample master mix, and the concentration range of the antibiotic standard curves was 0–100 ng/mL. The correlation coefficients of all calibration curves were greater than 0.99 (Table S1, Supplementary Information). The sensitivity and reproducibility are verified by inserting standard solutions in the sample sequence for every 10 samples. If the change in peak area is greater than 15%, then the new standard is reanalyzed, and subsequent samples are quantified according to the new calibration curve.

The limit of detection (LOD) is the lowest detectable concentration at a signal-to-noise ratio (S/N) of 3. The recovery was calculated using the spiked recovery method to verify the feasibility of the method. The recoveries of sulfonamide antibiotics, quinolone antibiotics, tetracycline antibiotics, and chloramphenicol antibiotics measured at a spiked concentration of 50 ng·L^−1^ were 67.10~91.45%, 62.31%~124.60%, 78.63~103.30%, and 77.00~128.33%, respectively, and the relative standard deviations (RSDs) of all analytes were less than 14%. Blank samples were used as quality control samples to verify whether the whole assay process was contaminated, and the results showed that the sample processing and onboard testing processes were not interfered with by contamination.

### 2.6. Ecological Risk Assessment 

The potential of contaminants to cause adverse effects was determined according to the risk quotient values (RQs) method in the European Commission’s technical guidance document. The values of RQs were estimated using Equation (1) [38] as follows:

RQ = MEC/PNEC,(1)

where MEC and PNEC were the measured environmental concentration and predicted no-effect concentration of each contaminant, respectively. The PNEC values for antibiotic toxicity data to aquatic organisms were obtained by searching the literature, as well as searching the US ETOCOX database, and the specific information is shown in Table 1.

In general, the levels of risk were classified into three groups: high risk (RQ > 1); medium risk (0.1 ≤ RQ ≤ 1); low risk (RQ < 0.1); no risk (RQ < 0.01) [39].

**Table 1:** Predicted no-effect concentrations (PNECs) of antibiotics.

Type of Antibiotic	Sensitive Species	PNEC (ng/L)	Reference
CIP	*M. aeruginosa*	17	[40]
DOC	*L. gibba*	316	[41]
TC	*P. subcapitata*	3310	[42]
OTC	*P. subcapitata*	1040	[43]
ENR	*M. aeruginosa*	49	ETOCOX Database
NOR	*M. aeruginosa*	32	[44]
OFL	*M. aeruginosa*	21	[40]
FLO	*C. dubia*	2300	[45]
ENO	Green alga	49	ETOCOX Database
TMP	*D. polymorpha*	29	[46]
STZ	*D. magna*	200	ETOCOX Database
SMX	*C. dubia*	210	[47]
SDZ	*S. capricomutum*	2200	[48]
PEF	NA	NA	NA
SUZ	*C. dubia*	18,900	ETOCOX Database
FLE	Green alga	11.3	ETOCOX Database
PIP	*C. dubia*	660,900	ETOCOX database
DAN	NA	NA	NA
OXO	*C. dubia*	467,740	ETOCOX Database
MNO	Green alga	530	ETOCOX Database
MAR	NA	NA	NA

NA indicates data is not yet available.

### 2.7. Statistical Analysis

SPSS 20.0 software (IBM, Armonk, NY, USA) and Origin Pro 2020 software (OriginLab, Northampton, MA, USA) were used to process the data. In this study, the significance was set at *p* < 0.05 level. If the data did not follow a normal distribution, non-parametric test was used for group comparisons, i.e., the Mann–Whitney U test and the Kruskal–Wallis H test were used for two groups and multiple groups, respectively. Spearman’s correlation test was used to test the correlation consumption. Before principal component analysis (PCA), the distribution of data of concentrations was determined. The log transformation was used to make data meet or approximately meet a normal distribution. For individual chemicals, half of its LOQ was selected if the concentration was less than its LOQ during statistical analysis.

## 3. Results and Discussion

### 3.1. Detection Frequency and Concentration of Antibiotics

**Figure 2:**
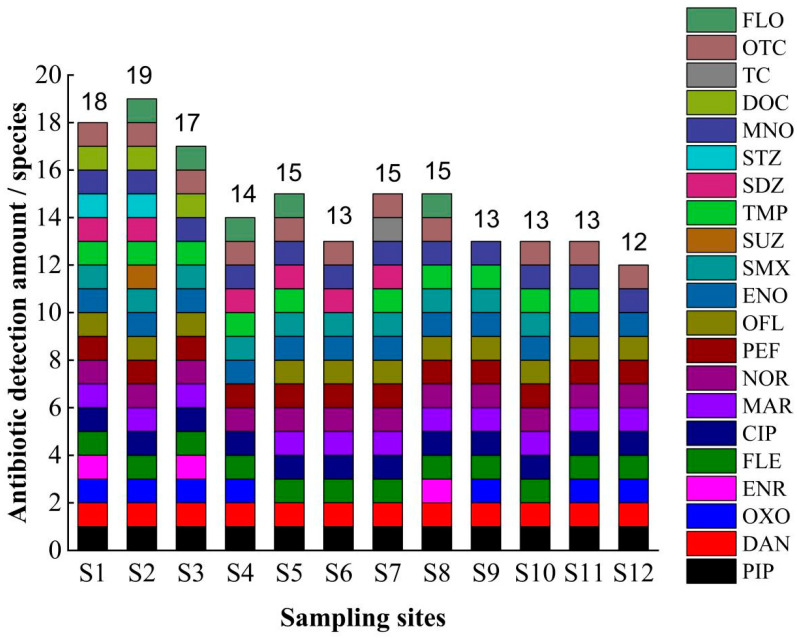
Detection characteristics of antibiotic compounds in seawater in the sampling area. Note: FLO is florfenicol; OTC is oxytetracycline; TC is tetracycline; DOC is doxycycline; MNO is minocycline; STZ is sulfathiazole; SDZ is sulfadiazine; TMP is sulfamethoxazole; SUZ is sulfamethoxazole; SMX is sulfamethoxazole; ENO is enrofloxacin; OFL is ofloxacin; PEF is pefloxacin; NOR is norfloxacin; MAR is meprofloxacin; CIP is ciprofloxacin; FLE is fleroxacin; ENR is enrofloxacin; OXO is oxalic acid; DAN is dalfloxacin; PIP is pyrimethamine.

In total, 50 antibiotics were analyzed, and 8 antibiotics, including PIP, DAN, FLE, CIP, NOR, PEF, ENO, and MNO, were detected at all sampling sites with quinolone antibiotics as well as tetracycline antibiotics as broad-spectrum antibiotics due to their high efficiency and long-lasting effect on antibacterial. These antibiotics are often widely used for sterilization in aquaculture, which could be the main reason for the detection of all eight antibiotics in this study area. The highest number of antibiotics was detected at sampling site S2, with 19 compounds. The complex geographical location of this sampling site, which is located at the mooring of fishing boats and surrounded by residential areas, as well as close to agricultural land and farming areas, may be the main reasons for the high number of antibiotics detected in this area. This implies that the use of antibiotics, geographical characteristics, hydrological characteristics, and physicochemical substances [49] all have some influence on the residues of antibiotics. In contrast, Zeng detected six antibiotics in the inland sea at two locations in Wanning Lingshui [50]. The use of antibiotics in this study area is more complex; MAR was detected only at sampling site S4, SUZ was detected only at sampling site S2, STZ was detected at sampling sites S1 and S2, DOC was detected at sampling sites S1, S2 and S3, TC was detected only at sampling site S7, and OFL was detected at all sampling sites except for sampling site S4. OTC was detected at all sampling sites except for sampling site S9 (Figure 2). These antibiotic residues may be related to the situation of the breeding industry in the sampling area and the degree of morbidity and human activities, as well as to the high quality and low cost of antibiotics and broad-spectrum antibacterial resistance.

**Figure 3:**
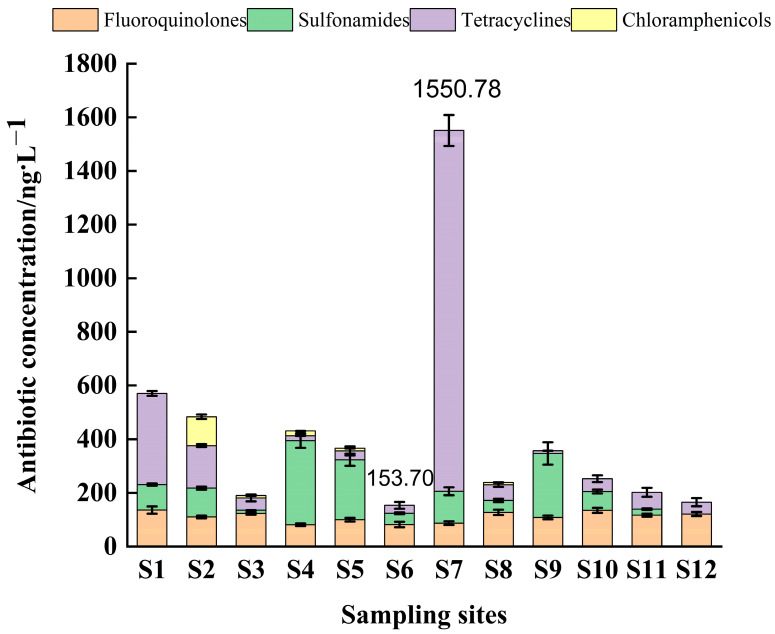
Total concentrations of antibiotics in seawater from different sampling sites.

**Figure 4:**
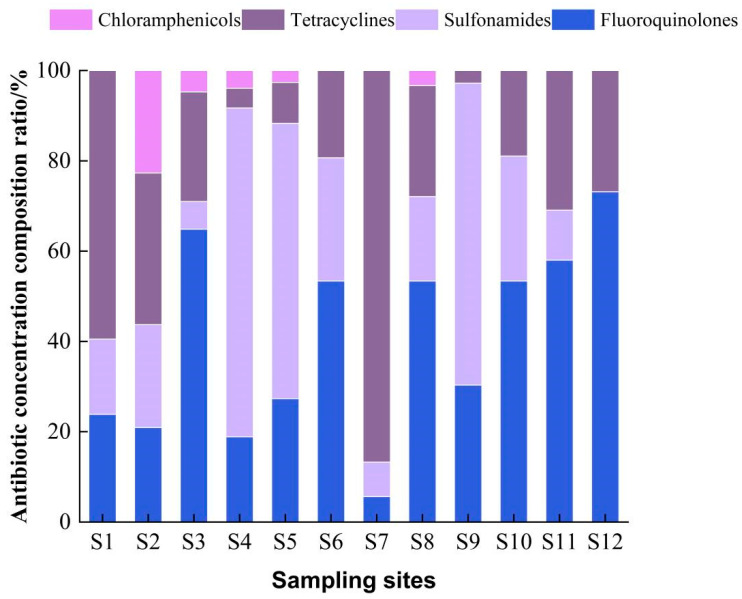
Proportion of antibiotics detected in seawater at different sampling sites.

According to Figure 3 and Figure 4, it can be seen that the highest concentration was detected at sampling site S7 with a total antibiotic level of 1550.8 ng/L; the lowest antibiotic detection concentration was at sampling site S12 with a total concentration of 153.6 ng/L; the total antibiotic concentration at the remaining sampling sites ranged from 160–550 ng/L. Compared with previous studies, the concentration of antibiotic residues in this study area was higher than that in the Guangzhou section of the Pearl River (dry season), and the highest antibiotic drug content was found in the Shenzhen River (1340 ng/L) [51], which was much higher than the total antibiotics detected in the nearshore seawater of Bohai Bay (24.3 to 242.7 ng/L), in the Bohai Sea (124.5–498.16 ng/L) [52], and also much higher than the antibiotic concentrations in nearshore seawater (10.28–156.63 ng/L) [38] in the eastern Hainan aquaculture area and Hainan Dongzhai port seawater (0.168–12.963 ng/L) [53], which may be related to the scale of aquaculture activities. The concentration of antibiotics varied among sampling sites, and the total concentration of tetracyclines was highest at sampling site S7 (1344.74 ng/L), followed by sampling sites S1 (339.11 ng/L) and S2 (158.26 ng/L), while the concentrations of tetracyclines at the remaining sampling sites were less than 100 ng/L.

The total concentrations of tetracyclines at sampling points S1 and S7 accounted for 62.83% and 86.24%, respectively. The elevated contamination of tetracycline antibiotics has been reported in major aquaculture waters around the coast of China. Similarly, in this study, the total concentration of tetracyclines was highest at site S7, accounting for 86.24%, which was similar to the results of the study in the nearshore waters of Bohai Bay, probably because this site is located near abandoned aquaculture ponds. Previous studies have shown that 3–20% of the total input flux of antibiotics in water comes from the diffusion of sediment to the aqueous phase. The discharge of culturing wastewater and the diffusion of antibiotics from the substrate may be the main reason for the high antibiotic concentrations in this region. The concentrations ranged from 20 to 240 ng/L at all sampling sites, except for sampling site S12, where no sulfonamides were detected. Studies have shown that sulfonamide antibiotics are not easily hydrolyzed and are easily photolyzed [18], indicating that the amount of sulfonamide antibiotics used at sampling site S12 was very small or even no sulfonamide antibiotics were used. The area is located offshore with long light hours and high intensity; it is possible that sulfonamide antibiotics in this area were photolyzed and thus not detected. While sampling site S4 had sulfonamide contamination with a percentage as high as 74.32%, which may be related to the geographical location of the sampling site and human activity. Moreover, sampling site S4 is a bay with poorly mobile water bodies, and the discharge of farm effluent from sampling sites S2 and S3 carried non-degradable sulphonamide antibiotics to sampling site S4, which may have resulted in the highest concentrations of sulphonamide antibiotics. Chloramphenicol had the highest concentration (106.89 ng/L) at sampling site S2, and these antibiotics are broad-spectrum that can treat a wide range of infectious bacterial diseases. The highest proportion of chloramphenicol (22. 48%) was detected at sampling site S2, which is located at the mooring of fishing boats and surrounded by residential areas, as well as close to agricultural fields, which may be the reason for the high concentration of chloramphenicol-like antibiotics in this area compared with other sampling sites. In addition, the use of chloramphenicol was banned in China in 1999, and chloramphenicol residues in this area may remain in the environment due to historical reasons [53]. The concentration of quinolones in all sampling sites ranged from 80–140 ng/L. The sampling site with the highest percentage of quinolones was S12 (68.23%), which may be due to the fact that quinolones are less easily hydrolyzed than other antibiotics.

The elevated antibiotic concentration in the sample collection area may be related to the scale of aquaculture and anthropogenic activities. Compared with other studies on mariculture areas, the concentration of antibiotic residues in nearshore seawater in Fengjia Bay was at a high level, which implies that the amount of antibiotics used in the area is high, and the sampling sites were placed relatively close to mangroves, which may also be due to the shade of trees near the mangroves resulting in lower light intensity and lower seawater temperature than other places, thus leading to high antibiotic residue concentrations [3]. The sampling sites were located relatively close to the mangroves, which may also be due to the weaker light intensity from the shade of the mangroves and the lower seawater temperature compared to other places, which is not conducive to the degradation of antibiotics, thus leading to the high concentration of antibiotic residues [4].

### 3.2. Possible Sources of Antibiotics and Correlation Analysis with Environmental Factors

**Figure 5:**
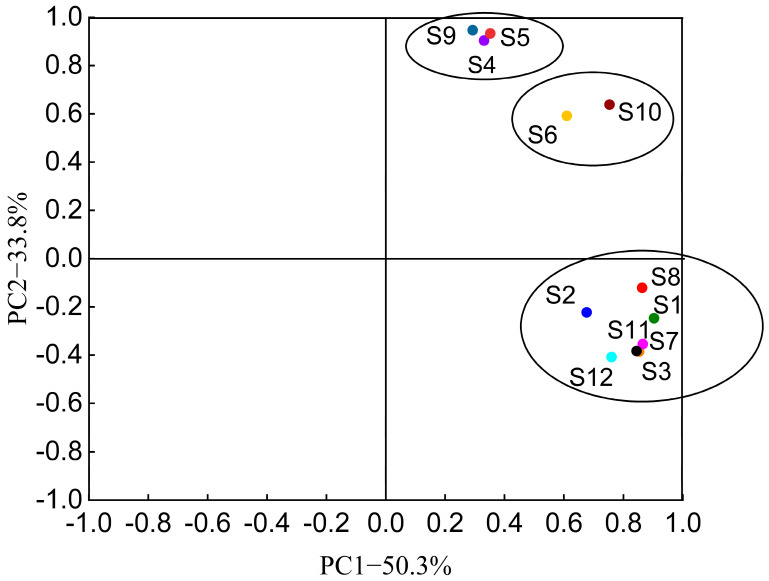
PCA analysis of antibiotics at each sampling site.

The first principal component, P1, explains 50.33% of the variance, the second component, P2, explains 33.77% of the variance, and the cumulative eigenvalue of P1 and P2 is 84.10%, principal component analysis (PCA) of the sample rotates component number is shown in Table 2. All sample points can be divided into three clusters (Figure 5). Among them, sampling points S4, S5, and S9 were one cluster, sampling sites S6 and S10 were one cluster, and sampling sites S1, S2, S3, S7, S8, S11, and S12 were one cluster. The clustered sampling sites indicated the similarity of antibiotic composition, indicating that the contamination sources of antibiotics in the sample sites might be similar. The distance between the three clusters was far, indicating that there may be different sources of antibiotic contamination. Combining the geographical location of the sampling sites and the surrounding environment, it is easy to find that cluster 1 and cluster 3 may be contaminated by different farming wastewater, which may be caused by the different species farmed in the two clusters, while in cluster 3, sampling sites S2 and S8 are farther away from other sampling sites, sampling site S2 is distributed around planting industry, and sampling site S8 has multiple pipeline sewage discharge, which may be the cause of the two sampling sites being farther away from other sampling sites in cluster 3. This may be the reason why the two sampling points are far away from other sampling points in cluster 3. The two sampling sites in cluster 2 are located at the shore of the nature reserve (S6) and the nearshore estuary where the vessels are moored (S10), and the area along the shore of the nature reserve is where the vessels pass by, and the vessels passing by may bring the residual antibiotics at sampling site S6 to sampling site S10, so the cluster may be due to human disturbance.

**Figure 6:**
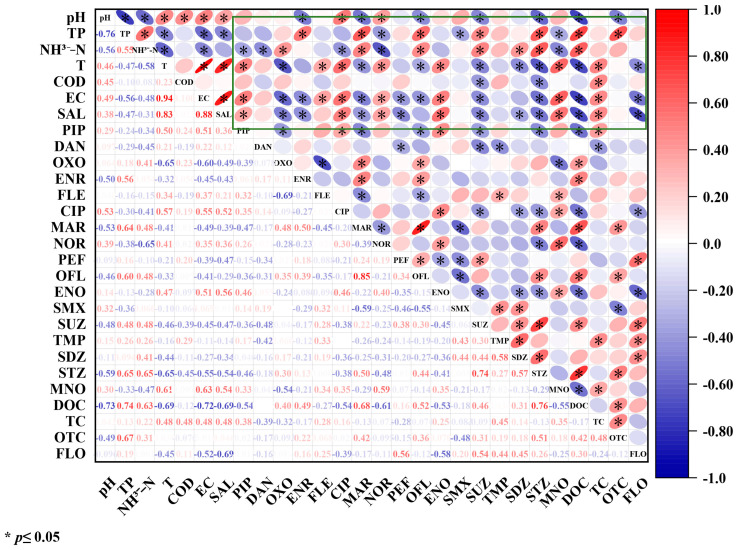
Spearman correlation analysis between antibiotics and environmental factors. Note: TP is total phosphorus, NH^3−^-N is ammonia nitrogen, T is temperature, COD is chemical oxygen demand, C-ms/cm is electrical conductivity, SAL is salinity; the green box area is the correlation between antibiotics and environmental factors.

The 21 antibiotics detected in the sampling area were correlated with the environmental factors, and according to Figure 6, it can be seen that there is some significant correlation between environmental factors and antibiotics; except for dissolved oxygen, all other environmental factors were significantly correlated with a variety of antibiotics, specifically: CIP and NOR were significantly positively correlated with pH, ENR, MAR, OFL, SUZ, STZ, DOC, while OTC was significantly negatively correlated with pH. The results of some studies showed that different antibiotics responded differently to pH in the case of 7.0 < pH < 9.0, the higher the pH, the faster the hydrolysis of antibiotics, thus changing the concentration of antibiotics in the aqueous environment. The total phosphorus was significantly positively correlated with ENR, MAR, OFL, SUZ, STZ, DOC, and OTC and negatively correlated with NOR, SMX, and MNO, which may be the result of antibiotics and feeds [54]. Ammonia nitrogen was significantly positively correlated with OXO, MAR, OFL, SDZ, STZ, and DOC and negatively correlated with PIP, DAN, CIP, NOR, and MNO. Further, the temperature was significantly negatively correlated with PIP, FLE, CIP, NOR, and MNO, significantly positively correlated with PIP, FLE, CIP, NOR, ENO, DOC, TC, and significantly negatively correlated with OXO, MAR, OFL, SUZ, SDZ, STZ, DOC, FLO. According to the literature [55], the higher the temperature, the faster the decomposition of antibiotics, and the temperature was negatively correlated with antibiotic residues. This present study also showed a similar trend to the literature results. Only a few antibiotics showed a positive correlation with temperature, which is possibly related to the strong stability of antibiotics and not easily affected by temperature; dissolved oxygen was only significantly positively correlated with TC and significantly negatively correlated with SUZ and STZ; conductivity was significantly positively correlated with PIP, FLE, CIP, NOR, ENO, MNO, and TC, and with OXO, ENR, MAR, PEF, OFL, SUZ, STZ, DOC, FLO, it was significantly negatively correlated; salinity was significantly positively correlated with PIP, CIP, NOR, ENO, MNO, TC and negatively correlated with OXO, ENR, MAR, PEF, SUZ, SDZ, STZ, DOC, and FLO. A previous study [56] proved that salinity can change the solubility of organic pollutants in water, and the differences in these properties may affect their distribution in the aqueous and sediment phases. The solubility of organic contaminants in water decreases with increasing salinity, and sediments are more likely to adsorb antibiotics under high salinity conditions, which leads to a decrease in antibiotic detection in the aqueous phase.

### 3.3. Environmental Risk Assessment

**Figure 7:**
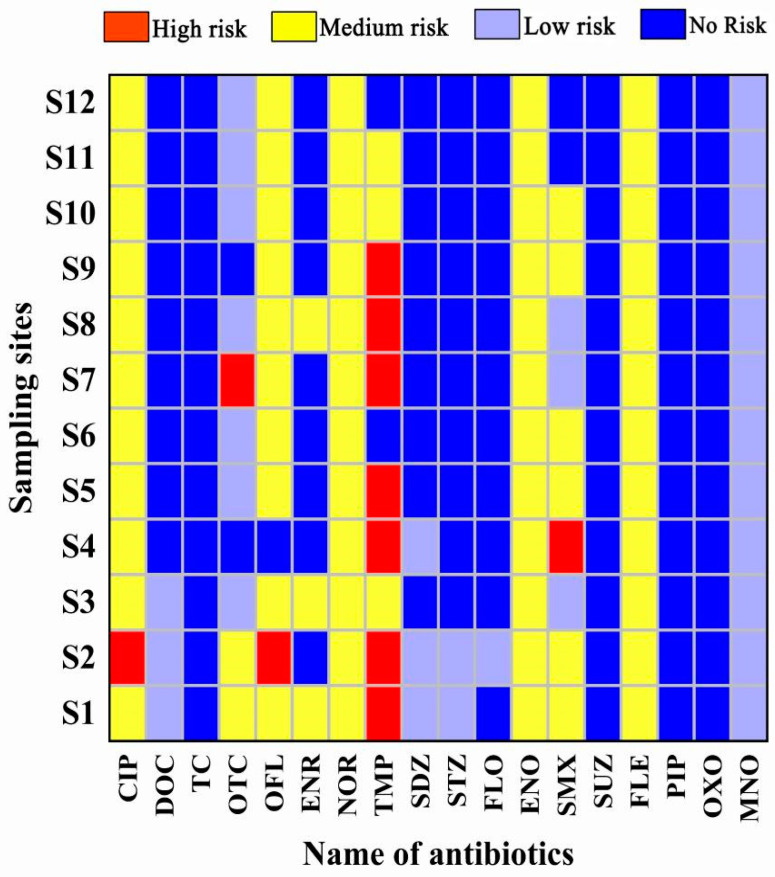
Ecological risk assessment grade of sampling sites.

According to the calculation method of risk quotient values, the RQ values of 18 antibiotics in different sampling points were derived, and the final ecological risk evaluation results are shown in Figure 7. It can be seen that most of the antibiotics in the sampling area showed no risk to the aquatic organisms in the sampling area, but the antibiotic CIP showed a high risk to *M. aeruginosa* in the sampling point S2 and a medium risk level in the remaining 11 sampling points. TMP antibiotic showed a high risk to *D. polymorpha* in the sampling points S1, S2, S4, S5, S7, S8, and S9. The antibiotic TMP showed a high risk for *D. polymorpha* in the sampling sites S1, S2, S4, S5, S7, S8, and S9, medium risk in the sampling sites S10 and S11, and no risk in the rest of the sites. OFL showed a medium risk for *M. aeruginosa* in the other 10 sampling sites except for no risk in S4 and high risk in S2. SMX showed a medium risk for *C. dioica* in 7 sampling sites. SMX showed a medium risk to *C. dubia* in 7 sampling sites, while the rest of the sites showed low or no risk. ENO, FLE, and NOR all showed a medium risk to the sensitive organisms in 12 sampling sites. It has been shown that antibiotic residues may cause acute or chronic toxic effects on aquatic organisms, and long-term antibiotic residues may stimulate the development of resistance in pathogenic bacteria and induce the production of resistance genes, which will certainly affect the stability of the ecosystem [57]. Several antibiotics in this study area have a high ecological risk to aquatic organisms and combined with the location of the sampling sites, it was found that the areas with medium-high risk to organisms are located in the farming area, which suggests that the discharge of farming wastewater may be the main reason for the medium-high risk of antibiotic performance to sensitive organisms in this study area. To address this phenomenon, firstly, the use of antibiotics and the discharge of farming and domestic wastewater should be regulated to reduce the use of CIP, NOR, TMP, OFL, ENO, SMX, and FLE in this area. At the same time, measures should be taken to reduce the discharge of antibiotics, such as developing antibiotic alternatives, etc. Meanwhile, the ecological risk of antibiotics to aquatic organisms in this study area should be monitored for a long time.

## 4. Conclusions

The antibiotics were detected in seawater from the Fengjiawan mariculture area in Wenchang City, with concentrations ranging from 153.7 to 1550.9 ng/L. The most detected antibiotics were quinolones, and the highest was tetracyclines. There was some variation in the antibiotic composition from one sampling site to another. Correlation analysis with environmental factors showed that pH, temperature, conductivity, salinity, NH^3^-N, and total phosphorus had a strong correlation with antibiotics. The ecological risk assessment using RQ values showed that most antibiotics in the sampling area had low ecological risk to aquatic organisms, and CIP, NOR, TMP, OFL, ENO, SMX, and FLE had some ecological risk. The ecological risk of CIP, NOR, TMP, OFL, ENO, SMX, and FLE to aquatic organisms in the sampling area is low and should be taken into account, and preventive measures should be taken. The ecological risk of antibiotics in the area should be monitored for a long time. This study is a reconnaissance investigation of antibiotics in the region, and due to the complex hazard of these emerging contaminants, more studies are needed to investigate the occurrence and ecotoxicological effects of these chemicals in the ocean.

## Figures and Tables

**Table 2:** Principal component analysis (PCA) of the sample rotates component number.

Sampling Sites	Rotated Component Number		Commonality (Common Factor Variance)
	1	2	
S1	**0.903**	−0.247	0.876
S2	**0.676**	−0.223	0.507
S3	**0.853**	−0.385	0.876
S4	0.331	**0.904**	0.926
S5	0.352	**0.933**	0.994
S6	**0.610**	**0.592**	0.722
S7	**0.865**	−0.354	0.873
S8	**0.863**	−0.121	0.759
S9	0.293	**0.947**	0.983
S10	**0.753**	**0.638**	0.973
S11	**0.844**	−0.383	0.859
S12	**0.760**	**−0.408**	0.743

Note: Bold indicates that the absolute value of the load factor is greater than 0.4.

## Data Availability

Not applicable.

## Supplementary Materials

The following supporting information can be downloaded at: https://www.mdpi.com/article/10.3390/toxics11050421/s1. Table S1: MRM parameters and standard curves for antibiotic analysis.
