# Supplementary material for: Occurrence and Distribution of Antibiotics in a Tropical Mariculture Area of Hainan, China: Implications for Risk Assessment and Management
Source: Toxics. 2023 May 1;11(5):421. doi: 10.3390/toxics11050421 (PMC10224144; doi:10.3390/toxics11050421)
Supplement: Supplementary file 1 [file toxics-11-00421-s001.zip › toxics-2313104-supplementary.pdf]

Table S1 MRM parameters and standard curves for antibiotic analysis

English name of antibiotics	Abbreviations	Q1	Q3	DP	CE	Standard Curve
Orbifloxacin	TCI	396	352	80	24	$y = 8.94024e^{4}$ x -21821.91607 (R^2 = 0.99941)
		396	295.2	80	32	
Pipemidic Acid	PIP	304.2	286.2	80	25	$y = 6.45010e^{4}$ x $-7.26468e^{5}$ (R^2 = 0.99803)
		304.2	217.1	80	31	
Danofloxacin	DAN	358.1	340.1	77	30	$y = 1.72249e^{5}$ x $-3.95064e^{6}$ (R^2 = 0.98491)
		358.1	314.1	77	24	
Oxolinic acid	OXO	262	244.1	70	26	$y = 6.82130e^{4}$ x + 4482.60440 (R^2 = 0.99884)
		262	216.1	70	40	
Enrofloxacin	ENR	360.1	316.1	80	28	$y = 1.07375e^{5}$ x $-1.06647e^{6}$ (R^2 = 0.99951)
		360.1	245.1	80	36	
Flumequin	FLU	262.1	244.1	77	23	$y = 1.54569e^{5}$ x + 12639.05199 (R^2 = 0.99921)
		262.1	202.1	77	42	
Fleroxacin	FLE	370	326.1	80	27	$y = 4.77205e^{4}$ x $-5.27761e^{5}$ (R^2 = 0.99908)
		370	269.2	80	35	
Ciprofloxacin	CIP	332.2	231	115	50	y = 3.31473e4 x $-3.99354e^{5}$ (R^2 = 0.99701)
		332.2	288.1	115	26	
Lomefloxacin	LOM	352	265	80	33	y = 8.02757e4 x + -2.82282e5 (R^2 = 0.99838)
		352	308.1	80	28	
Marbofloxacin	MAR	363.1	320.1	80	23	y = 4.64285e4 x + -4.11989e5 (R^2 = 0.99713)
		363.1	72	80	46	
Nalidixic acid	NAL	233	215	68	18	y = 9.06446 x + -23.07866 (R^2 = 0.99420)
		233	187	68	34	
Norfloxacin	NOR	320.1	302.3	140	27	y = 23341.23907 x + -2.86223e5 (R^2 = 0.99801)

		320.1	276.1	140	24	
Pefloxacin	PEF	334.1	316.1	80	27	y = 5.87854e4 x + -7.68300e5 (r = 0.99740)
		334.1	290.2	80	25	
Sarafloxacin	SAR	386.2	342.3	100	25	y = 7.04559e4 x + -3.86021e5 (r = 0.99949)
		386.2	299	100	38	
Difloxacin	DIF	400.1	356.1	80	28	y = 8.89290e4 x + -3.65391e5 (r = 0.99831)
		400.1	299.1	80	41	
Sparfloxacin	SPA	393	349.2	80	30	y = 7.13468e4 x + -3.11537e5 (r = 0.99830)
		393	292	80	38	
Cinoxacin	CIN	263.1	217.1	60	30	y = 7.81813e4 x + 7.63706e4 (r = 0.99911)
		263.1	245	60	22	
Ofloxacin	OFL	362.3	318	140	26	y = 3.82777 x + -26.17728 (r = 0.99293)
		362.3	261	140	38	
Enoxacin	ENO	321	303	80	24	y = 19382.27416 x + -2.55796e5 (r = 0.99558)
		321	234	80	30	
sulfaguanidine	SGN	215	108	45	33	y = 6574.36797 x +· -1137.65126 (r = 0.99918)
		215	156	45	21	
Sulfamonomethoxine	SMM	281.1	156	75	25	y = 27313.67611 x + -6.23530e4 (r = 0.99796)
		281.1	126.1	75	30	
Sulfaphenazole	SPZ	315	156	90	27	y = 7602.15002 x + -9116.64017 (r = 0.99790)
		315	108	90	40	
Sulfabenzamide	SML	277.1	156	60	19	y = 5556.30405 x + -2960.49409 (r = 0.99901)
		277.1	108	60	32	
Sulfapyridine	SP	250.3	184.3	60	25	y = 5.04606e4 x + -5.42272e4 (r = 0.99941)
		250.3	156.1	60	23	
sulphacetamide	SCT	215	156	52	17	y = 8665.96588 x + 1981.44157 (r = 0.99774)

		215	108	52	29	
Sulfameter	SM	281.2	215.3	60	25	y = 3.99794e4 x + -1.02087e5 (r = 0.99753)
		281.2	156	60	25	
Sulfamoxole	SMO	268.1	156.1	82	22	y = 11864.27026 x + -4.98844e4 (r = 0.99400)
		268.1	113.2	82	25	
Sulfamethazine	SDD	279.2	186.1	45	24	y = 26127.17727 x + -26705.78507 (r = 0.99998)
		279.2	124.2	45	33	
Sulfadimethoxine	SDT	311.1	156.1	120	28	y = 10985.89562 x + -23605.21681 (r = 0.99840)
		311.1	108.2	140	47	
Sulfisoxazole	SFZ	268.1	156.1	82	22	y = 8613.86568 x + -17904.40539 (r = 0.99885)
		268.1	113.2	82	25	
Sulfisomidin sodium	SM2	279.2	186.1	70	23	y = 4.77166e4 x + -9.26982e4 (r = 0.99027)
		279.2	124.4	70	30	
Sulfamerazine	SMZ	265.2	172.1	70	23	y = 3.17758e4 x + -4.97038e4 (r = 0.99898)
		265.2	156.1	70	23	
Sulfamethoxazole	SMX	254.1	156	65	22	y = 25024.98229 x + -26878.81956 (r = 0.99926)
		254.1	108	65	36	
Sulfamethizol	SUZ	271	156.2	30	19.3	y = 22092.77515 x + 20654.18070 (r = 0.99683)
		271	108	30	35	
Sulfamethoxydiazole	TMP	291.1	230.1	60	33	y = 1.20452e5 x + 17771.34695 (r = 0.99998)
		291.1	123.1	95	34	
Sulfadoxine	SD	311.1	156.1	70	30	y = 6.84920e4 x + -5.74194e4 (r = 0.99919)
		311.1	108.2	70	37	
Sulfamethoxypyridazine	SMP	281	126.1	75	27	y = 8.24300e4 x + -2.15302e5 (r = 0.99233)
		281	156	75	25	
Sulfachinoxalin	SQ	301.1	156	120	22	y = 8226.04871 x + -10095.24078 (r = 0.99589)

		301.1	92.2	120	40	
Sulfachloropyridazine	SCP	285.1	156.1	21	20.3	y = 8174.75435 x + -11749.90166 (r = 0.99613)
		285.1	92	21	39.2	
Sulfadiazine	SDZ	251.1	156	40	22	y = 22989.63498 x + -6.95711e4 (r = 0.99900)
		251.1	92	40	32.1	
Sulfathiazole	STZ	256.1	156.1	38	21	y = 4.11978e4 x + -9.44579e4 (r = 0.99722)
		256.1	108.1	38	31	
Sulfanitran	APNPS	336.1	156.1	60	22	y = 1127.37928 x + 871.17006 (r = 0.99981)
		336.1	294	60	22	
Chlortetracycline	CTC	479.2	462	120	24	y = 18094.13999 x + -8.08714e4 (r = 0.99814)
		479.2	444.1	120	31	
Minocycline	MNO	458.2	441.2	130	25	y = 18922.01479 x + -1.97066e5 (r = 0.99847)
		458.2	352.2	130	40	
Doxycycline	DOC	445.2	428.1	140	27	y = 2386.70360 x + -9036.47859 (r = 0.99866)
		445.2	410.2	140	34	
Tetracycline	TC	445.2	427.3	120	19	y = 2724.20877 x + -4069.08738 (r = 0.99856)
		445.2	410.3	120	27	
Oxytetracycline	OTC	461.3	443.2	100	19	y = 17835.89835 x + -7.16758e4 (r = 0.99988)
		461.3	426.2	100	27	
Florfenicol	FLO	356	336	-40	-13	y = 14716.04272 x + 4.71798e4 (r = 0.99663)
		356	185	-40	-26	
Thiamphenicol	THI	353.9	289.9	-75	-18	y = 8.97666e4 x + 22245.19261 (r = 0.99928)
		353.9	184.9	-75	-28	
Chloramphenicol	CHP	321	257.1	-40	-15	y = 3.17541e4 x + 1.11640e5 (r = 0.99724)
		321	152	-40	-24	
